# Effect of Enzymatically Extracted Fucoidans on Angiogenesis and Osteogenesis in Primary Cell Culture Systems Mimicking Bone Tissue Environment

**DOI:** 10.3390/md18090481

**Published:** 2020-09-21

**Authors:** Julia Ohmes, Yuejun Xiao, Fanlu Wang, Maria Dalgaard Mikkelsen, Thuan Thi Nguyen, Harald Schmidt, Andreas Seekamp, Anne S. Meyer, Sabine Fuchs

**Affiliations:** 1 Experimental Trauma Surgery, University Medical Center Schleswig-Holstein, 24105 Kiel, Germany; Julia.ohmes@uksh.de (J.O.); xiao_yuejun@163.com (Y.X.); Fanlu.wang@uksh.de (F.W.); Andreas.seekamp@uksh.de (A.S.); 2 Protein Chemistry and Enzyme Technology Section, DTU Bioengineering, Department of Biotechnology and Biomedicine, Technical University of Denmark, Building 221, 2800 Kongens Lyngby, Denmark; mdami@dtu.dk (M.D.M.); thuthi@dtu.dk (T.T.N.); asme@dtu.dk (A.S.M.); 3 MetaPhysiol, Am Römerberg, 55270 Essenheim, Germany; schmidt@metaphysiol.de

**Keywords:** fucoidan, angiogenesis, osteogenesis, *Fucus evanescens*, outgrowth endothelial cells, mesenchymal stem cells, VEGF, angiopoietin, enzyme-assisted extraction, sulfation degree

## Abstract

Angiogenesis, the formation of new blood vessels from existing ones, is an essential process for successful bone regeneration. Further, angiogenesis is a key factor for the development of bone-related disorders like osteosarcoma or arthritis. Fucoidans, sulfated polysaccharides from brown algae, have been shown to affect angiogenesis as well as a series of other physiological processes including inflammation or infection. However, the chemical properties of fucoidan which define the biological activity vary tremendously, making a prediction of the bioactivity or the corresponding therapeutic effect difficult. In this study, we compare the effect of four chemically characterized high molecular weight fucoidan extracts from *Fucus distichus* subsp. *evanescens* (FE_crude and fractions F1, F2, F3) on angiogenic and osteogenic processes in bone-related primary mono- and co-culture cell systems. By determining the gene expression and protein levels of the regulatory molecules vascular endothelial growth factor (VEGF), angiopoietin-1 (ANG-1), ANG-2 and stromal-derived factor 1 (SDF-1), we show that the extracted fucoidans negatively influence angiogenic and osteogenic processes in both the mono- and co-culture systems. We demonstrate that purer fucoidan extracts with a high fucose and sulfate content show stronger effects on these processes. Immunocytochemistry of the co-culture system revealed that treatment with FE_F3, containing the highest fucose and sulfate content, impaired the formation of angiogenic tube-like structures, indicating the anti-angiogenic properties of the tested fucoidans. This study highlights how chemical properties of fucoidan influence its bioactivity in a bone-related context and discusses how the observed phenotypes can be explained on a molecular level—knowledge that is indispensable for future therapies based on fucoidans.

## 1. Introduction

Angiogenesis, the formation of new blood vessels from existing ones, is indispensable for bone regeneration and maintenance of bone health [1,2,3]. Whether for bone healing after trauma, improving bone health during systemic diseases or for controlling tumor growth during osteosarcoma, angiogenesis has become a promising target in bone-related disorders [4,5,6]. Blood vessels deliver nutrients, minerals and oxygen to the damaged tissue, but they also serve as niches and guiding structures for bone and blood progenitor cells [7,8]. Various signaling molecules like angiopoietins and growth factors carefully regulate the angiogenic homeostasis. Vascular endothelial growth factor (VEGF), one of the most important pro-angiogenic mediators, induces migration of endothelial cells and permeability of the endothelial cell layer in blood vessels. Angiopoietin-1 and -2 (ANG-1, ANG-2) regulate the endothelial transition from a quiescent into an activated state. The quiescent state is characterized by highly interconnected endothelial cells with strong survivability. In the activated state, angiogenesis is initiated by endothelial cell migration and a more permeable endothelial cell layer [9]. Stromal-derived factor 1 (SDF-1), another important factor during angio- and osteogenesis functions as a chemoattractant for circulating osteogenic and osteoclast precursor, as well as endothelial cells. Attracted cells become relevant building elements for active angiogenic and osteogenic processes [10].

Fucoidan, a sulfated polysaccharide from the cell wall of brown algae and other marine invertebrates, has attracted increasing attention amongst researchers, because of the plethora of reported bioactivities. Fucoidans have shown to act on coagulation [11], angiogenesis [12], osteogenesis [13] and inflammation [14] amongst others. Fucoidans from *Undaria pinnatifida* and *Fucus vesiculosus* were already “Generally Recognized As Safe (GRAS)” for the use in food by the Food and Drug Administration (FDA) in the USA. Additionally, the ionic nature of fucoidan and its biodegradability turn this marine molecule into a promising candidate for biomaterials in the field of tissue engineering and regenerative medicine [15]. However, the chemical structure of fucoidan, which defines the biological activity, depends on many factors like species, timepoint of harvesting and extraction method [16,17]. Varying chemical properties explain contradictory reports of bioactivities like pro- and anti-angiogenic [18]. Insufficiently chemically characterized fucoidans impede the comparability of studies and prevent a clear definition of structure–bioactivity relationship. The chemical properties of fucoidan for medical applications must be reproducible in order to guarantee the desired activity and omit undesired side effects. Therefore, reproducible extraction techniques are needed, as well as more insights about specific bioactivities of chemically well-characterized extracts.

To better understand how fucoidans affect angio- and osteogenic processes on a molecular level in a bone tissue setting and what influence chemical properties have, we treated mono- and co-cultures of primary endothelial and osteoblast-like cells with four chemically well-characterized fucoidan extracts and quantified expression and protein levels of the important angiogenic regulators VEGF, SDF-1, ANG-1 and ANG-2. Additionally, we quantified alkaline phosphatase (ALP) activity and calcification level as early and late osteogenic differentiation markers, respectively. Therefore, we isolated human outgrowth endothelial cells (OEC) from blood [19] and mesenchymal stem cells from bone tissue [20] and differentiated the mesenchymal stem cells to an osteoblast-like lineage (MSC). We extracted crude fucoidan (FE_crude) from *Fucus distichus* subsp. *evanescens* (FE) with a green and reproducible enzyme-assisted extraction. The extraction method uses enzymes that cleave specific cell wall components and release fucoidan in its native form [21]. The crude fucoidan extract was purified into three fractions (FE_F1, FE_F2, FE_F3) using ion-exchange chromatography. The high molecular weight (HMW) fucoidans FE_crude, FE_F1, FE_F2 and FE_F3 differed mainly in mono-saccharide content and degree of sulfation. We treated OEC and MSC mono-cultures and co-cultures of both cell types for seven days with the extracts. We found that all extracts negatively influence angiogenic and osteogenic processes. We show that the anti-angiogenic and anti–osteogenic effect was stronger with purer extracts that contain a higher fucose and sulfate content (FE_F2 and F3). We demonstrate that expression and protein levels of the angiogenic mediator molecules VEGF, SDF-1, ANG-1 were downregulated in the MSC mono-culture and ANG-2 was downregulated in the OEC mono-culture. Expression levels in the co-culture were not affected by fucoidan treatment. Even though tube formation was clearly suppressed in the co-culture, VEGF protein levels were slightly increased after fucoidan treatment.

This paper (a) describes the bioactivity of four chemically well-characterized fucoidan extracts on angio- and osteogenesis in a mono- and co-culture system mimicking bone tissue, (b) provides information about the relationship between reported activities and chemical properties of the extracts, and (c) discusses a possible mechanism of action of HMW fucoidans in the context of angiogenesis in the mono- and co-culture systems.

## 2. Results

### 2.1. Chemical Properties of Enzymatically Extracted Fucoidans FE_Crude, F1, F2 and F3

The fucoidans were isolated by an enzyme-assisted extraction method [21]. The crude fucoidan (FE_crude) containing 59 % alginate, mainly mannuronic acid (ManA) of low molecular weight of 2-3 kDa, was fractionated by anion-exchange chromatography to obtain the three fractions FE_F1, FE_F2 and FE_F3. FE_F1 contains 34% fucose and 32% mannuronic acids, while F2 and F3 are considered pure fucoidans with 75% and 88% fucose, respectively, and 0% mannuronic acids.

^1^H NMR revealed specific signals for fucose (anomeric, ring and methyl protons) in all fractions. A characteristic peak for uronic acids (glucuronic acid (GluA), ManA) however was only detected in the fraction FE_F1. The ^1^H NMR spectra gave indications for 1→3, as well as 1→4 glycosidic linkages. The spectra and further details can be found in the previous publication by Nguyen and colleagues [21].

The fucoidans in FE_crude have a molecular weight main peak around 400 kDa. The molecular weight of fucoidans in FE_F1 is more heterogeneous with the main peak around 400 kDa and with a smaller peak at 30–40 kDa. Both FE_crude and F1 have additional peaks around 2–3 kDa indicating alginate impurities. The molecular weight of the fucoidans in F2 ranges from 12–800 kDa with the main peak at 400–800 kDa and a shoulder around 40 kDa. Fucoidans in F3 have a more homogenous size with the main peak at 600 kDa.

The sulfate content of the extracts increases with their purity. FE_crude, F1, F2 and F3 have a sulfate content of ~22, ~20, ~35 and ~39%, respectively.

The total phenolic content of the extracts decreases with their purity. FE_crude, F1, F2 and F3 have a total phenolic content of 5.2, 2.9, 3.0 and 0.8 mgGAE per g dry weight, respectively.

The total protein content is equal or less than 0.15% for all extracts and therefore negligible. The chemical properties of the tested extracts are displayed in Table 1.

### 2.2. Tolerance of Primary OEC and MSC towards Enzymatically Extracted Fucoidans

**Figure 1:**
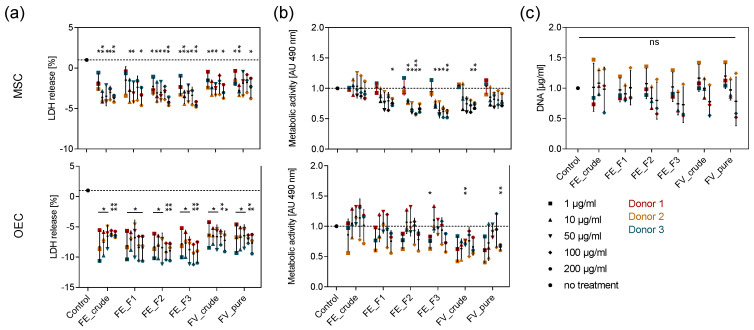
Effect of fucoidan concentration on the membrane integrity and metabolic activity in MSC and outgrowth endothelial cells (OEC). Mesenchymal stem cells (MSC) and OEC were treated with five different fucoidan concentrations ranging from 1–200 µg/mL. LDH release as an indicator for membrane integrity (**a**) and metabolic activity determined by MTS assay (**b**) were quantified after seven days of treatment. (**c**) MSC were treated with 1, 10 and 100 µg/mL fucoidan. The DNA content was quantified after seven days of treatment. The mean values of experiments with three individual donors ± s.e.m. were plotted. All values were normalized to the control. Significances compared to the control were calculated with Welch’s *t*-test (* *p* < 0.05, ** *p* < 0.01, *** *p* < 0.001).

To test the tolerance of OEC and MSC towards different doses of the enzymatically extracted fucoidans, we studied the metabolic activity and membrane integrity of the cells after seven days of treatment with five different fucoidan concentrations ranging from 1 to 200 µg/mL using MTS and LDH assays, respectively. Fucoidan treatment did not increase the release of LDH in both cell types, indicating that none of the extracts is cytotoxic (Figure 1a). The metabolic activity of OEC was not affected by fucoidan treatment, while it was lowered by treatment with the purer FE_F2 and FE_F3 extracts in MSC. A concentration-dependent trend can be observed, as high fucoidan concentrations decrease metabolic activity in MSC more prominently (Figure 1b). To assess whether these findings are related to a decreased MSC proliferation or are only related to metabolic effects, we quantified the DNA content in the respective MSC mono-cultures. The quantification of DNA content in MSC confirms that the cell number is not significantly altered by treatment with any tested fucoidan extract (Figure 1c). These results demonstrate that all tested doses are non-toxic to primary OEC and MSC and ensure that differences in protein production are not reduced to an unequal number of cells.

### 2.3. Influence of Fucoidan Extracts on Angiogenic Mediators in OEC and MSC Mono-Culture

**Figure 2:**
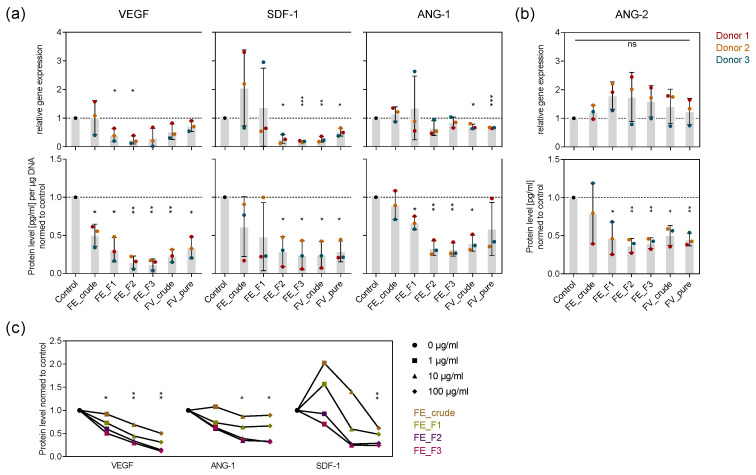
Effect of fucoidan extracts on angiogenic mediators in OEC and MSC mono-culture. MSC (**a**) and OEC (**b**) were treated with 100 µg/mL fucoidan for seven days. The expression and protein level of angiogenic markers vascular endothelial growth factor (VEGF), stromal-derived factor 1 (SDF-1), angiopoietin-1 (ANG-1) and ANG-2 were quantified with qPCR and enzyme-linked immunosorbent assay (ELISA), respectively. The mean values of experiments with three individual donors ± s.e.m. were plotted. (**c**) MSC were treated with 1, 10 and 100 µg/mL fucoidan for seven days. Protein levels of VEGF, SDF-1 and ANG-1 were quantified with ELISA. The mean values of experiments with three individual donors were plotted for every extract. Trend lines visualize the influence of concentration on protein level for each extract. All plotted values were normalized to the control. Significances compared to the control were calculated with Welch’s *t*-test (* *p* < 0.05, ** *p* < 0.01, *** *p* < 0.001).

To examine how signaling molecules mediating angiogenic processes are influenced by the tested fucoidans in mono-culture and which role the chemical composition of fucoidan plays, we treated OEC and MSC for seven days with the extracts and quantified the expression and protein level of the angiogenic mediators VEGF, SDF-1, ANG-1 (MSC), and ANG-2 (OEC) in the respective mono-culture systems. The expression of VEGF and SDF-1 was downregulated after fucoidan treatment in MSC, while ANG-1 expression in MSC and ANG-2 expression in OEC was not affected (Figure 2a,b top). Fucoidan treatment decreased the level of all the mentioned proteins (Figure 2a,b bottom). The fractions FE_F3 and F2 decreased marker expression and production to the highest extent, followed by FE_F1 and FE_crude, respectively (Figure 2a,b). Additionally, 100 µg/mL treatment decreased the protein level of VEGF, SDF-1 and ANG-1 in MSC mono-culture the most, followed by 10 and 1 µg/mL, respectively (Figure 2c). The experiment demonstrates that the tested fucoidans impair key angiogenic regulators in OEC and MSC mono-culture. Purer fucose-rich fucoidans possessing a high sulfate content, such as FE_F3 and FE_F2, as well as high concentrations, such as 100 µg/mL, downregulate angiogenic mediators more effectively.

### 2.4. Influence of Fucoidan Extracts on Osteogenic Markers in MSC Mono-Culture

**Figure 3:**
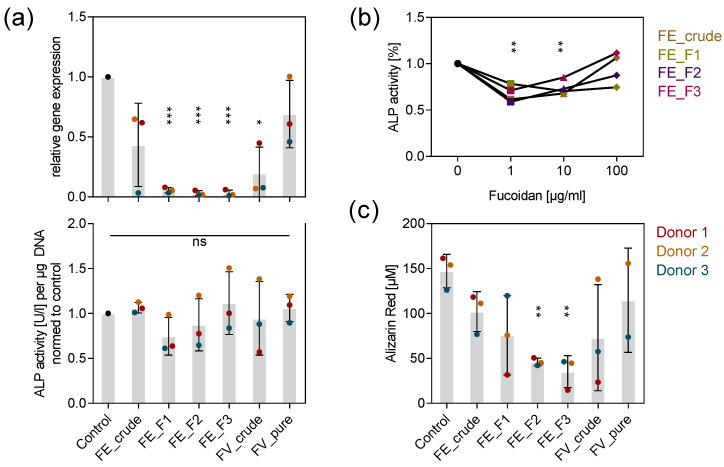
Effect of fucoidan extracts on osteogenic markers in MSC mono-culture. (**a**) MSC were treated with 100 µg/mL fucoidan for seven days. Expression of early osteogenic marker ALP was quantified by qPCR (top). ALP activity was determined as an indicator of protein abundance (bottom). (**b**) MSC were treated with 1, 10 and 100 µg/mL fucoidan for seven days. ALP activity was quantified and the mean values of experiments with three individual donors were plotted for every extract. Trend lines visualize the influence of concentration on ALP activity for each extract. (**c**) MSC were treated with 100 µg/mL fucoidan for 14 days. The calcification level as a late osteogenic marker was quantified by alizarin red staining. The mean values of experiments with three individual donors ± s.e.m. were plotted. Significances compared to the control were calculated with Welch’s *t*-test (* *p* < 0.05, ** *p* < 0.01, *** *p* < 0.001).

To assess whether osteogenic processes, which are tightly coupled to angiogenesis, were similarly impaired by the tested extracts, we quantified the expression and activity of the early osteogenic marker ALP after seven days and determined the calcification level as a late osteogenic differentiation marker after 14 days. Treatment with the purer fractions FE_F1, FE_F2 and FE_F3 decreased ALP expression almost completely (Figure 3a top). Interestingly, the treatment of 100 µg/mL fucoidan did not affect the ALP activity (Figure 3a bottom). However, lower fucoidan concentrations, such as 1 and 10 µg/mL decreased ALP activity approximately by 30% (Figure 3b). Alizarin red staining was used as an indicator of calcium deposition during osteogenic mineralization. All tested fucoidans lowered the calcification level in MSC, although the purer fucoidans FE_F2 and FE_F3 impaired calcium deposition with a reduction of 60–70% to the highest extent (Figure 3c). The results indicate that early and late osteogenic events are affected by fucoidan treatment. Unlike the impact of fucoidans on angiogenesis, lower concentrations decreased the amount of active ALP more effectively. Similarly to the activity of fucoidan described for angiogenesis, extracts with a high fucose and sulfate content affected osteogenic processes stronger than crude ones, as shown for ALP expression and the deposition of calcium.

### 2.5. Visualization and Quantification of Angiogenic Structures in MSC-OEC Co-Culture

**Figure 4:**
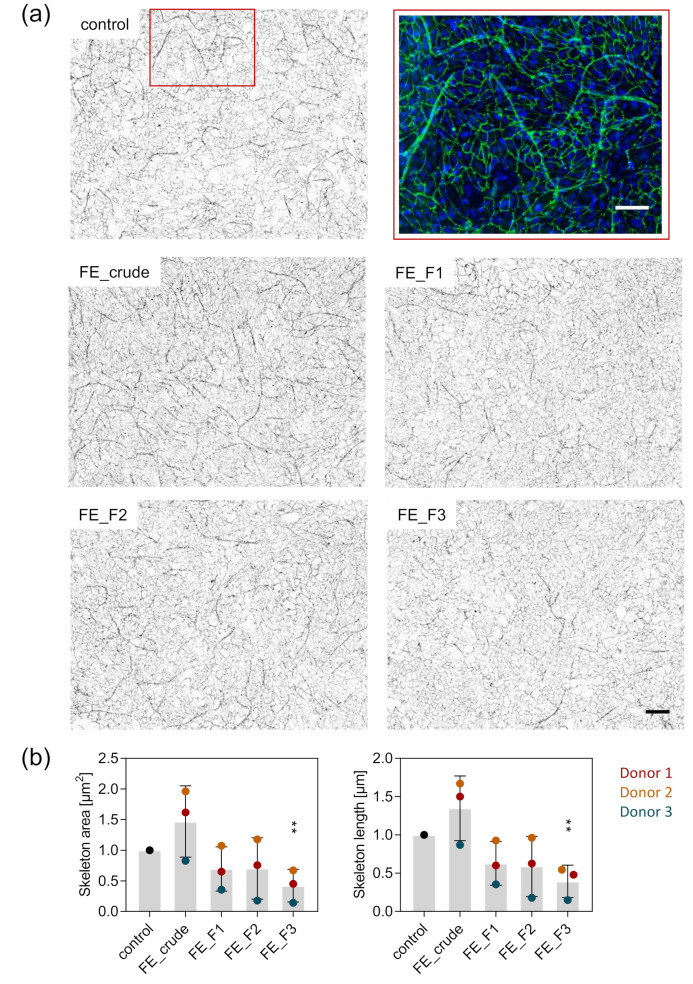
Effect of fucoidan on the formation of angiogenic tube-like structures in OEC-MSC co-culture. OEC and MSC were co-cultured and treated with 10 µg/mL fucoidan for seven days. (**a**) The cells were fixed and stained for the endothelial cell-specific molecule VE-cadherin and nuclei. A close-up of the formed angiogenic tube-like structures is shown in the red frame (VE-cadherin in green, nuclei in blue, scale bar = 100 µm). The overview images were inverted and displayed in grey values (scale bar = 200 µm). (**b**) Area and length of the angiogenic skeleton were quantified semi-automatically. Three stitched images consisting of nine frames were quantified for each donor. The mean values of experiments with three individual donors ± s.e.m. were plotted. Significances compared to the control were calculated with a *t*-test (** *p* < 0.01).

To visualize the influence of the fucoidan extracts on the formation of angiogenic tube-like structures, we co-cultured OEC and MSC and stained for the endothelial cell-specific adherens junction molecule VE-cadherin one week after fucoidan treatment. In the co-culture system, cells are able to interact, giving OEC the possibility to establish first angiogenic structures. Subsequently, we quantified the length and area of the emerged tube-like structures. Treatment with the fucoidan fractions FE_F1, F2 and F3 impaired the formation of these structures. The observed effect was strongest when cells were treated with the fraction FE_F3, richest in fucose and sulfates (Figure 4a). The quantification of structure length and area supports the observations from the microscopy pictures. Structure length and area were decreased by treatment with the fucoidan fractions FE_F1 and F2 to the same extent, while FE_F3 treatment decreased structure length and area significantly.

### 2.6. Influence of Fucoidan Extracts on Angiogenesis in OEC-MSC Co-Culture

**Figure 5:**
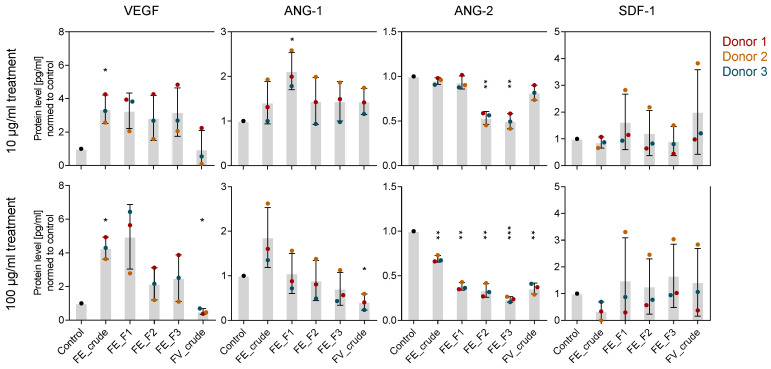
Effect of fucoidan extracts on angiogenic mediators in MSC-OEC co-culture on a molecular level. MSC-OEC co-cultures were treated with 10 and 100 µg/mL fucoidan for seven days. Protein levels of pro-angiogenic markers VEGF, ANG-1, ANG-2 and SDF-1 were quantified by ELISA. The mean values of experiments with three individual donors ± s.e.m. were plotted. Significances compared to the control were calculated with Welch’s t-test (* *p* < 0.05, ** *p* < 0.01, *** *p* < 0.001).

To reveal more details on how fucoidans interfere with the angiogenic process on a molecular level, we determined expression and protein level of the angiogenic mediators VEGF, ANG-1, ANG-2 and SDF-1 in OEC-MSC co-culture after 7 days of treatment. In contrast to the mono-culture system, all the tested marker genes were expressed as in the control cells (Figure S2). While ANG-2 protein levels were decreased similarly to the mono-culture system, VEGF protein levels were slightly increased (Figure 5). Furthermore, ANG-1 protein levels were slightly decreased when cells were treated with 100 µg/mL of FE_F2 and FE_F3. Differences in protein levels due to the extract purity occurred only when cells were treated with a high fucoidan concentration of 100 µg/mL. As observed in the mono-culture, treatment with purer extracts lowered protein levels of VEGF, ANG-1 and ANG-2 in a stronger way (Figure 5 bottom).

## 3. Discussion

In this study, we examined four HMW fucoidan extracts (FE_crude: ~400 kDa, FE_F1: ~40 and ~400 kDa, FE_F2: ~40 and ~600 kDa, FE_F3: ~600 kDa), differing mainly in monosaccharide content and sulfation degree, in regard to their effect on angio- and osteogenesis in primary OEC and MSC. Furthermore, we provide data to reveal details about the molecular mechanism that underlies the observed effects. OEC and MSC in mono- and co-culture were treated with four different fractions from enzyme-assisted extracted fucoidans from *F. evanescens*. The extraction technique releases fucoidan likely preserving its native structural conformation. The obtained crude fucoidan molecules were fractionated by ion-exchange chromatography, also removing remaining sugar impurities like alginate oligosaccharides [21]. Based on MTS and LDH data presented in this study, we can exclude the toxicity of the tested extracts and further determine concentrations ranging from 10-100 µg/mL as appropriate for the following experiments (Figure 1). By quantifying the expression and protein levels of four prominent angiogenic regulatory molecules (Figure 2, Figure 5) and visualizing the tube formation in the co-culture systems (Figure 4), we found that the extracts inhibit angiogenesis-related processes and associated key regulatory molecules in primary OEC and MSC.

Angiogenesis is a very dynamic and carefully regulated process. To better understand the molecular mechanism underlying the observed anti-angiogenic phenotype caused by fucoidan treatment, we quantified VEGF, SDF-1, ANG-1 and ANG-2 expression and protein level in mono- and co-cultures (Figure 2 and Figure 5).

For the mono-culture system, we show that fucoidan downregulates VEGF and SDF-1 gene expression in MSC and decreases the protein levels of ANG-2 in the OEC and VEGF, SDF-1 and ANG-1 in the MSC cell culture supernatant (Figure 2). Results from the OEC-MSC co-culture allow insights to the mechanism of action of fucoidan when both cell types are able to communicate, reflecting physiological processes in a more realistic way. For the co-culture, we show that fucoidan treatment decreases ANG-2 protein levels but slightly increases VEGF protein levels in the cell culture supernatant (Figure 5). Despite the trend of increased VEGF levels, tube formation was impaired by fucoidans in the co-culture system (Figure 4) suggesting that the presence of the tested fucoidans blocks VEGF-mediated downstream signaling events in endothelial cells needed for angiogenic activation.

Various studies already claimed that fucoidans interfere with the formation of blood vessels. However, due to different experimental set-ups and fucoidans, the results of these studies vary tremendously from anti- [12,22,23,24,25,26] to pro-angiogenic [27,28,29,30] and yet it is not finally clarified which chemical requirements a fucoidan extract must meet to achieve specific bioactivities.

Angiogenesis is a key regulator of healthy bone homeostasis and a prerequisite for osteogenesis [8,31]. To reveal whether osteogenic processes, next to angiogenesis, were also directly influenced by the different tested fucoidans from *F. evanescens*, we quantified ALP expression and activity as an early and the degree of calcification as a late indicator for osteogenic differentiation. We found that the tested fucoidans negatively influence osteogenic processes in MSC mono-culture (Figure 3). VEGF, produced by mesenchymal stem cells and osteoblasts, is one of the most important regulatory molecules for angiogenesis, but it also promotes osteoblast differentiation [31]. By triggering angiogenesis and the release of osteogenic cytokines such as BMP from endothelial cells, VEGF regulates recruitment of bone progenitor cells and osteoblast differentiation in a paracrine manner [7,32]. Additionally, to an autocrine regulation [33,34], Liu et al. propose an intracrine mechanism, where intracellular VEGF-VEGF-receptor complexes enter the nucleus and activate transcription factors that promote osteoblast differentiation [35]. Thus, a decreased VEGF level due to fucoidan treatment could explain the reduced osteoblast differentiation (Figure 3a,b) and calcium deposition (Figure 3c) in MSC mono-culture, as well as the reduced metabolic activity with increasing fucoidan concentrations (Figure 1b top) in accordance to the above-mentioned mechanisms.

According to literature, fucoidan can affect bone health by influencing osteoblast differentiation as well as bone resorption [36,37], both processes that depend on VEGF as a central regulatory molecule. In contrast to our observation, a number of studies show that fucoidans promote osteoblast differentiation [38,39,40]. However, most studies examined the effect of LMW fucoidan (<15 kDa), while we report on the bioactivity of HMW fucoidans (>350 kDa). Another important aspect potentially influencing the biological effect of fucoidans and possibly explaining controversial reports in the literature is how fucoidans are presented to the model system. We applied fucoidan as a free molecule in solution to the cell culture systems, whereas a lot of studies investigated the bioactivity of fucoidans incorporated in a delivery system. As reported for other sulfated sugars [41,42], also fucoidans seem to be able to bind growth factors like VEGF and their receptors. Supporting the assumption that fucoidans are able to bind to VEGF, we showed in an ELISA-based competitive binding assay that the tested extracts suppress the binding of VEGF to its antibody in a concentration-dependent manner (Figure S1). The binding capacity of fucoidan to biological factors like VEGF supposedly lowers growth factor concentration when fucoidan is applied in solution to cell culture systems. Incorporated into a delivery system, however, the affinity of fucoidan to bioactive molecules can be used to achieve a local enrichment of growth factors such as VEGF. In the context of a biocomposite implant material or scaffold, the binding capacities of fucoidan could be beneficial for bone tissue engineering approaches [43,44,45].

The affinity of fucoidan to regulatory molecules seems crucial for explaining different observed bioactivities. Additionally to the molecular weight [46,47] and the way how fucoidans are applied, the sulfation degree of the molecule and the monosaccharide composition seem to have an essential impact on the biological effect [18,48] In this study we compare crude fucoidan extracts containing uronic acids, a low fucose and sulfate content (FE_crude, F1) with pure extracts containing no alginate impurities and a high fucose and sulfate content (FE_F2, F3). Literature reports that sulfates are located mainly at the 2-position of fucose residues in fucoidan from *Fucus evanescens* [49]. Consistently, the chemical analyses revealed that pure extracts with high fucose content such as FE_F2 and F3 also have a high sulfate content and vice versa. ^1^H NMR of the fucoidan fractions confirmed the presence of alginate impurities in FE_F1 and revealed that the polysaccharides consist of 1→3 and 1→4 glycosidic linkages. We show that fucose-rich fucoidans with a high sulfate content enhance the anti-angiogenic and -osteogenic effect (Figure 2, Figure 3a top, Figure 3c, Figure 5 bottom), concluding that pure fucoidan extracts with an increased sulfate content are favorable in order to achieve high bioactivity in the tested system. In accordance with our observations, Soeda and colleagues demonstrated that chemically oversulfated fucoidan from *Fucus vesiculosus,* unlike its native counterpart, reduced the formation of capillary-like structures in HUVECS [50]. Similarly to Haroun-Bouhedja and colleagues [51], we hypothesize that a higher density of sulfates increases the negative charge of the fucoidan molecule, hence facilitating the formation of fucoidan–protein complexes. Lake and colleagues compared the effect of LMW fucoidan (~5 kDa) on VEGF_165_ that contains a heparan sulfate binding site and on VEGF_121_ lacking the binding motif for the sulfated sugar. They found that fucoidan is only able to enhance VEGF_165_-driven chemotaxis of endothelial cells, highlighting the importance of sulfates for the bioactivity of fucoidans. It becomes evident that sulfates play a crucial role in the bioactivity of fucoidans. However, observed effects cannot be reproduced using other sulfated polysaccharides like chondroitin sulfate [52] or heparin [53]. Thus, other chemical properties like molecular weight distribution might play a role in the bioactivity of fucoidan in addition to the sulfate content. It is worth mentioning that co-extracted compounds like proteins or polyphenols could also influence the observed bioactivity. For the tested extracts, however, the protein content is too small for a significant contribution to the biological effect (Table 1). Treatment with the pure fucoidan fractions FE_F2 and F3 achieved the highest observed bioactivity. Considering the low polyphenolic content in these extracts (Table 1), it can be concluded that the observed effect results very likely from fucoidan and not from co-extracted polyphenols.

**Figure 6:**
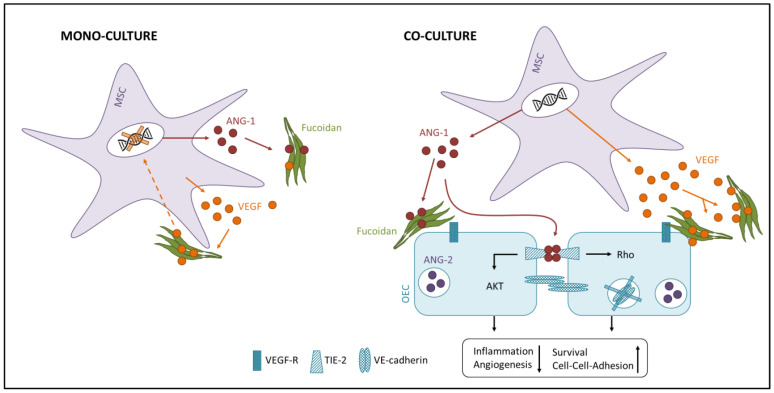
Hypothetical anti-angiogenic molecular mechanism of high molecular weight (HMW) fucoidan in the mono- and co-culture system. The interaction of fucoidan with VEGF and angiopoietins and/or endothelial cells might block the bioactivity of the respective signaling molecules or intracellular signaling cascades and cause the observed anti-angiogenic phenotype.

Concluding, we hypothesize based on the data that fucoidan treatment causes OEC to switch from an activated into a quiescent state. Although still unclear, this effect might be caused by the binding of fucoidan to VEGF and/or endothelial cells, hence blocking bioactivity of VEGF or intracellular downstream signaling pathways. In the quiescent state, ANG-1 regulates the stabilization and survival of existing angiogenic structures, while the migration of OEC and formation of new blood vessels is suppressed [9] (Figure 6 right). ANG-2 is increasingly released by endothelial cells upon pro-angiogenic stimuli [54]. Consistently, we detected lower protein levels of ANG-2 in the fucoidan-treated co-cultures. The mono-culture system reflects angiogenic processes only in a limited way. Nevertheless, fucoidan acts probably similarly in the mono-culture as in the co-culture and causes the observed effects by interacting with signaling molecules like VEGF and angiopoietins. Feedback mechanisms due to locally concentrated signaling molecules might explain the downregulated expression of the respective genes (Figure 6 left).

In this study, we described the effect of four chemically well-described HMW fucoidans from *Fucus distichus* subsp. *evanescens* on angio- and osteogenesis in primary OEC and MSC mono- and co-culture systems. We found that the tested fucoidans exhibit anti-angiogenic and anti-osteogenic properties in these test systems. We analyzed the impact of purity and sulfation degree on bioactivity and found that purer extracts with a higher sulfate content increase the anti-angiogenic and anti-osteogenic effect probably due to their interaction with signaling molecules like VEGF and angiopoietins.

Clearly, fucoidans hold promising bioactivities for different medical fields. The fucoidans tested in this study might be applied in a bone-related context to limit angiogenesis which is a critical factor for bone tumor formation, metastasis or inflammation.

## 4. Materials and Methods

### 4.1. Ethical Approval

All experiments with primary cells from human tissue were performed with the consent of the donors and were approved by the local ethical advisory board.

### 4.2. Isolation of Fucoidan from Algae

#### 4.2.1. Algal Material

Over two years old *Fucus distichus* subsp. *evanescens* was collected in March 2017 from 1 m water depth at the Kiel Canal, Germany. The upper 2/3 part was harvested and kindly provided by Coastal Research and Management GmbH.

#### 4.2.2. Fucoidan Extraction from *Fucus Distichus* Subsp. *evanescens*

Before the extraction, *Fucus distichus* subsp. *evanescens* brown algae was washed, lyophilized and grounded into a powder. The enzymatic extraction was performed as described in [21]. Briefly, dried seaweed was treated with Cellic^®^CTec2 cellulase (Novozymes, Bagsværd, Denmark) and alginate lyase SALy from *Sphingomonas* sp. to break down non-fucoidan polysaccharide components of the cell and release fucoidan. High molecular weight alginate was removed by precipitating with CaCl_2_. Fucoidans were further precipitated using ethanol. The crude fucoidan product was lyophilized for bioactivity studies (FE_crude). The aqueous solution of the crude fucoidan was further purified and fractionated using ion-exchange chromatography, obtaining three fractions (FE_F1, F2, F3) as previously described in [21]. Subsequently, the fractions were filtered through a 10 kDa membrane and lyophilized.

### 4.3. Analysis of Chemical Properties of Crude and Fractionated Fucoidan

The performance and detailed results of most chemical analyses of the fucoidan extracts are described in [21]. Shortly, high-performance size exclusion-chromatography was used to determine the molecular weight distribution of the extracts. Sulfate content was quantified using the BaCl_2_ gelatin method [55]. Monosaccharide content was determined using high-performance anion-exchange chromatography HPEAC-PAD with pulsed amperometric detection. ^1^H NMR was performed with the Avance III-700 and with the Avance III-500 HD NMR spectrometer as specified in [21].

The total phenolic content of the tested extracts was determined by the Folin–Ciocalteu method as described in [53]. Briefly, 20 µL of aqueous fucoidan sample (40–50 mg/mL) were mixed with 200 µL Folin–Ciocalteu reagent. After 5 min of incubation, 30 µL Na_2_CO_3_ were added and incubated for another 2 h. Subsequently, the absorbance was measured and the total phenolic content was expressed as gallic acid equivalents (GAE) in mg per g of dry fucoidan weight.

The total protein content of the tested extracts was determined with the Bradford assay using BSA as a standard [56].

### 4.4. Cell Cultivation

#### 4.4.1. Isolation and Expansion of OEC

Human outgrowth endothelial cells (OEC) were isolated from human peripheral blood according to the previously published protocol [19]. In brief, mononuclear cells were isolated from buffy coats by gradient centrifugation using Biocoll (Biochrom, Berlin, Germany) and resuspended in Endothelial Basal Medium 2 (EBM-2) (Promocell, Heidelberg, Germany) including all Endothelial Growth Medium 2 (EGM-2) associated supplements (Promocell), 5% FBS (Sigma-Aldrich, Steinheim, Germany) and 1% Penicillin/Streptomycin (PS) (Biochrom). Resuspended cells were seeded in collagen type I-coated (Corning, Bedford, MA, USA) 24-well plates at a density of 5 × 10^6^ cells/cm^2^. After one week cells were detached by trypsination and sub-cultured in new collagen type I-coated 24-well plates at a density of 5 × 10^5^ cells/cm^2^. Colonies of cobblestone-shaped OEC grew within two to three weeks. Obtained OEC were cultivated in fibronectin-coated (Millipore, Temecula, CA, USA) plates or flasks in EGM-2 including 7% FBS and 1% PS. The medium was exchanged every second day and cells were sub-cultured every three to four days when confluent.

#### 4.4.2. Isolation and Expansion of MSC

Human mesenchymal stem cells derived from cancellous bone were isolated from femoral heads according to the previously published protocol [20]. In brief, bone segments were washed in phosphate-buffered saline (PBS) and detached cells were collected by centrifugation. Cells were resuspended in Dulbecco’s Medium Essential Medium (DMEM)/Ham’s F-12 (Biochrom) including 20% FBS and 1% PS and seeded into collagen type I-coated flasks at a density of 2 × 10^6^ cells/cm^2^. After the first sub-culture, the FBS content in the medium was reduced from 20% to 10%. Mesenchymal stem cells from passage number two were cultured in osteogenic differentiation medium (ODM: DMEM/Ham F-12 supplemented with 10% FBS, 1% PS, 50 µM L-ascorbic acid 2-phosphate (Sigma-Aldrich), 10 mM β-glycerophosphate (Sigma-Aldrich) and 0.1 µM dexamethasone (Sigma-Aldrich)) to obtain osteoblast-like differentiated cells (MSC). The medium was exchanged every second day.

### 4.5. Fucoidan Treatment in MSC and OEC Mono-Culture

MSC or OEC were seeded in 96- or 24-well plates at a density of 40,000 cells/cm^2^. On the next day, the culture medium was replaced by fresh medium containing the specific fucoidan concentrations. To keep the sampling time-point consistent for gene expression, protein level determination and immunocytochemistry staining, most experiments were harvested after seven days of treatment. For these experiments, the medium was refreshed on the third treatment day. Therefore, the old medium was replaced by fresh medium containing the same fucoidan concentrations as before. All experiments were performed with cells from three individual donors. Passage numbers of MSC and OEC ranged from 4–5.

### 4.6. Control Cells and Reference Substances

Control cells were cultured in medium without fucoidan. For a better comparability and as a reference substance, commercially available fucoidan from *Fucus vesiculosus* from Sigma Aldrich (FV_crude (F5631) and FV_pure (F8190)) was included in the experiments.

### 4.7. MTS and LDH Assay

MSC or OEC were treated with 1, 10, 50, 100 or 200 µg/mL fucoidan. The LDH release as well as the metabolic activity of OEC and MSC were determined after one, three and seven days of fucoidan treatment. Pierce LDH Cytotoxicity Assay Kit (Thermo Scientific, Rockford, IL, USA) was used following the manufacturer’s protocol. The metabolic activity was assayed with the CellTiter 96 AQueous One Solution Cell Proliferation Assay (Promega, Madison, WI, USA) following the manufacturer’s protocol.

### 4.8. DNA Quantification

MSC were treated for seven days with 1, 10 and 100 µg/mL fucoidan. For harvesting, cells were washed with PBS and collected by scraping. The cell membrane was destroyed by freeze–thaw cycles and sonication. The released dsDNA content was quantified with the Quant-iT^TM^ PicoGreen^TM^ dsDNA Assay Kit (Invitrogen, Eugene, OR, USA) in a 96-well plate. Therefore, 100 µL TE-buffer, followed by 72 µL PicoGreen reagent were added per well. Then, 28 µL sample or standard were added per well and incubated for 10 min at 200 rpm. The fluorescence was measured at 485 nm excitation and 535 nm emission wavelength.

### 4.9. Quantification of Alkaline Phosphatase Activity in MSC

MSC were treated for seven days with fucoidan. The medium was collected and activity of ALP was measured using the Alkaline Phosphatase Assay Kit (Colorimetric) (abcam, Cambridge, UK) according to the manufacturer’s protocol. In brief, samples were applied to a 96-well plate, followed by addition of ALP substrate *p*nPP. Absorbance of the converted p-Nitrophenol was measured at 405 nm with a microplate reader after 1 h incubation at 25 °C.

### 4.10. Alizarin Red Staining for Determination of Calcification Level

MSC were treated for 14 days with fucoidan. Cells were fixed with 4% paraformaldehyde in PBS followed by the addition of 1 mL 40 mM Alizarin Red S Stain Solution (Millipore) to each well. After 30 min of incubation, cells were washed with distilled water until rinsed water became clear. To extract the bound dye, 600 µL 10% cetylpyridinium chloride (CPC) (Carol Roth GmbH, Karlsruhe, Germany) was added to each well and incubated overnight. An amount of 150 µL CPC including the extracted dye was transferred to wells of a 96-well plate and the absorbance was measured at 560 nm. Samples were compared to a standard of alizarin red dilutions in CPC.

### 4.11. Fucoidan Treatment in MSC-OEC Co-Culture

MSC were seeded in fibronectin-coated plates at a density of 40,000 cells/cm^2^ on the first day. On the second day, OEC were seeded at the same density on top of the MSC. Cells were cultured in EGM-2 (7% FBS, 1% PS). On the next day, the culture medium was replaced by fresh EGM-2 containing the specific fucoidan concentrations. For cell experiments that were assayed or harvested after seven days, the medium was refreshed on the third treatment day. Therefore, the old medium was replaced by fresh medium containing the same fucoidan concentrations as before. All experiments were performed with cells from three different donors. Passage numbers of MSC and OEC ranged from 4 to 5 and from 4 to 8, respectively.

### 4.12. Immunocytochemistry of Angiogenic Structures in MSC-OEC Co-Culture

MSC and OEC were co-cultured on fibronectin-coated Thermanox^®^ coverslips (Nunc, Rochester, NY, USA) in 24-well plates. After seven days of fucoidan treatment, angiogenic tube-like structures emerged and were stained for VE-cadherin. Therefore, cells were fixed with 4% PFA in PBS for 15 min, permeabilized with 0.5% Triton^TM^ X-100 (Sigma-Aldrich) for 15 min and unspecific binding sites were blocked with 1% BSA in PBS for 30 min. Human VE-cadherin primary antibody (AF938 R&D) was applied in a concentration of 4 µg/mL in 1% BSA in PBS for 1 h after 3x washing with PBS for 5 min. AlexaFluor488-linked secondary antibody (A11055 Invitrogen) was applied in a concentration of 2 µg/mL in 1% BSA in PBS for 1 h after repeated washing. Nuclei were stained with Hoechst 33258 in a concentration of 2 µg/mL. Samples were mounted on objective slides using Fluoromount^TM^ (Sigma-Aldrich) and imaged with a fluorescence microscope.

### 4.13. Quantification of Gene Expression by Quantitative Real-Time PCR

MSC/OEC mono- or co-cultures were treated for seven days with fucoidan. Cell lysates were generated by replacing the culture medium with 100 µL RNA Lysis Buffer T per well and incubating the cells for 10 min at 37 °C. Then, cell lysates were collected and RNA was isolated with the peqGOLD Total RNA kit (VWR, Leuven, Belgium), including DNA digestion via DNase I (VWR) treatment, according to the manufacturer’s protocol. An amount of 1 µg RNA was transcribed to cDNA using the High-Capacity RNA-to-cDNA^TM^ Kit (Applied Biosystems, Vilnius, Lithuania) following the manufacturer’s protocol. After transcription, the volume was filled up to 100 µL with nuclease-free water and gene expression of VEGF, SDF-1, ANG-1, ANG-2 and ALP was determined with quantitative real-time PCR. Therefore, 3.2 µL cDNA was mixed with 16.8 µL master mix containing 10 µL SYBR^TM^ Select Master Mix (Applied Biosystems), 2 µL QuantiTect Primer Assays (Qiagen, for further details, see Table 2) and 4.8 µL nuclease-free water. RPL13A was used as a housekeeping gene. DNA was amplified with a two-step program (heating to 50 °C for 2 min, heating to 95 °C for 2 min, 40 cycles of 95 °C for 15 s and 60 °C for 60 s) and relative gene expression was calculated using the ΔΔC_t_ method. All values were normed to the expression level of the control cells.

### 4.14. Enzyme-linked Immunosorbent Assay (ELISA) for Protein Level Quantification

MSC/OEC mono- or co-cultures were treated for seven days with fucoidan. The medium was collected and protein levels of VEGF, ANG-1, ANG-2 and SDF-1 were quantified using DuoSet^®^ ELISA Development Systems (R&D Systems, Minneapolis, MN, USA) according to the manufacturer’s protocol.

### 4.15. Image Analysis of Angiogenic Structures

OEC-MSC co-cultures were treated with fucoidan for seven days. Cells were fixed and stained for VE-cadherin as described above. For each condition, three stitched microscopy pictures were taken, each consisting of nine single frames. Before quantifying the area and length of the angiogenic skeleton, the background of the microscopy pictures was subtracted, followed by a shading correction and frame stitching using the BASiC [57] and Grid/Collection Stitching [58] plug-in, respectively, in FIJI [59]. Angiogenic skeleton area and length were quantified semi-automatically using the software ImageJ Version 1.42 [60] as described in a previous publication [61].

### 4.16. Statistical Analysis

The mean values ± standard deviation from three independent experiments were plotted. Statistical significances were calculated with the Welch’s t-test or t-test using GraphPad Prism 7.03. Values were considered statistically significant when *p* < 0.05.

## Figures and Tables

**Table 1:** Chemical analyses of *Fucus distichus* subsp. *evanescens* (FE) crude and fractionated extracts F1, F2 and F3. Further details regarding the chemical properties of these extracts can be found here [21].

Chemical Analyses		FE_Crude	FE_F1	FE_F2	FE_F3
Monosaccharide content [%mol]	Fucose	24.8 ± 2.9	34 ± 3.1	74.7 ± 0.8	87.8 ± 1.4
	Glucose	0.7 ± 0.1	7.7 ± 0.7	1.4 ± 0.1	0.3 ± 0.1
	GluA	1.0 ± 0.2	3.8 ± 0.3	0.3 ± 0.0	0.5 ± 0.1
	ManA	58.4 ± 2.6	32.2 ± 0.6	0.2 ± 0.0	0.0 ± 0.0
Sulfate content	Sulfate (SO_4_^2-^) [%]	21.7 ± 0.5	20.4 ± 3.4	34.8 ± 2.0	38.7 ± 1.0
	Degree of sulfation [weight% ratio SO_4_^2−^:Fuc]	1.4	0.8	1.0	1.0
Molecular weight	Range [kDa]	12–800 *	12–800 *	12–800	110–800
	Peak MW [kDa]	~400	~40 and ~400	~40 and ~600	~600
Total phenolic content [GAE/g]		5.19 ± 0.07	2.93 ± 0.11	3.02 ± 0.02	0.76 ± 0.04
Total Protein content [%]		≤0.15	≤0.15	≤0.15	≤0.15

* contain an additional alginate oligosaccharide peak at ~2–3 kDa.

**Table 2:** QuantiTect Primer Assays used in the presented study.

Gene	QuantiTect Primer ASSAY	Catalogue Number
Vascular Endothelial Growth Factor A (VEGF)	Hs_VEGFA_2_SG	QT01036861
Stromal-derived Factor 1 (SDF-1/CXCL12)	Hs_CXCL12_1_SG	QT00087591
Angiopoietin 1 (ANG-1)	Hs_ANGPT1_1_SG	QT00046865
Angiopoietin 2 (ANG-2)	Hs_ANGPT2_1_SG	QT00100947
Alkaline phosphatase (ALP)	Hs_ALPL_1_SG	QT00012957
60S ribosomal protein L13a (RPL13A)	Hs_RPL13A_1_SG	QT00089915

## Supplementary Materials

The following are available online at https://www.mdpi.com/1660-3397/18/9/481/s1, Figure S1: Interaction of fucoidan with VEGF and/or VEGF antibody, Figure S2: Effect of fucoidan extracts on the expression of pro-angiogenic marker genes in OEC-MSC co-culture.
