# Supplementary material for: Effect of Enzymatically Extracted Fucoidans on Angiogenesis and Osteogenesis in Primary Cell Culture Systems Mimicking Bone Tissue Environment
Source: Mar Drugs. 2020 Sep 21;18(9):481. doi: 10.3390/md18090481 (PMC7550999; doi:10.3390/md18090481)
Supplement: Supplementary file 1 [file marinedrugs-18-00481-s001.pdf]

SUPPLEMENTS

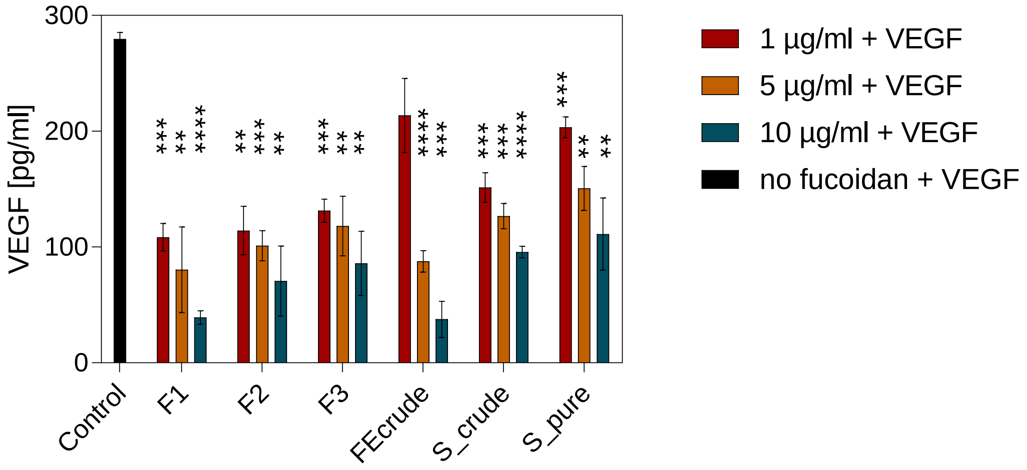

Figure S1. Interaction of fucoidan with VEGF and/or VEGF antibody. VEGF DuoSet® ELISA Development Systems (R&D Systems, Minneapolis, MIN, USA) was used according to the manufacturer's protocol. 300 µg/ml VEGF was mixed with 1, 5 and 10 µg/ml fucoidan extract 30 min before application. The control solution only contained 300 µg/ml VEGF. The mean values of the technical replicates ± s.e.m. were plotted. Significances compared to the control were calculated with Welch's t-test (*p<0.05, **<0.01, ***p<0.001, ****p<0.0001).

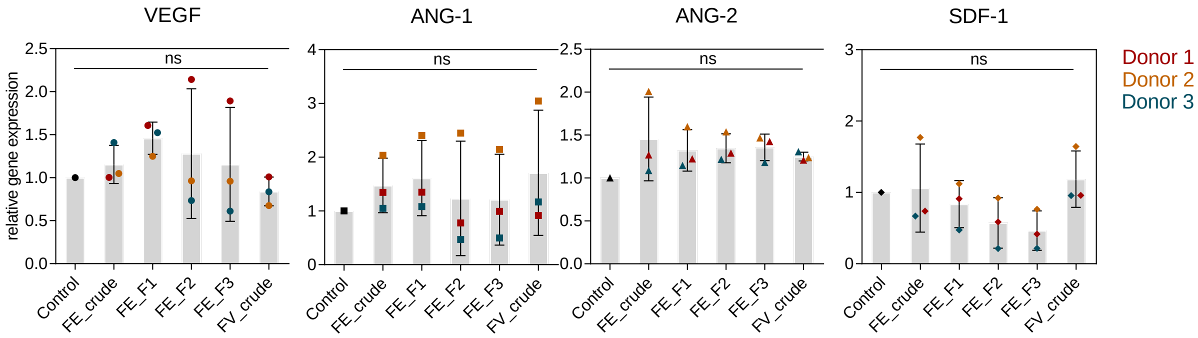

Figure S2. Effect of fucoidan extracts on the expression of pro-angiogenic marker genes in OEC-MSC co-culture. MSC-OEC co-culture was treated with 100 µg/ml fucoidan for seven days. The expression of VEGF, ANG-1, ANG-2 and SDF-1 was quantified with qPCR. The mean values of experiments with three individual donors ± s.e.m. were plotted. All plotted values were normalized to the control. Significances compared to the control were calculated with Welch's t-test (ns=not significant).
